# *STAT4*, *TRAF3IP2*, *IL10*, and *HCP5* Polymorphisms in Sjögren's Syndrome: Association with Disease Susceptibility and Clinical Aspects

**DOI:** 10.1155/2019/7682827

**Published:** 2019-02-10

**Authors:** Serena Colafrancesco, Cinzia Ciccacci, Roberta Priori, Andrea Latini, Giovanna Picarelli, Francesca Arienzo, Giuseppe Novelli, Guido Valesini, Carlo Perricone, Paola Borgiani

**Affiliations:** ^1^UOC Reumatologia, Dipartimento di Medicina Interna e Specialità Mediche, Sapienza Università di Roma, Italy; ^2^Department of Biomedicine and Prevention, Genetics Section, University of Rome Tor Vergata, Italy; ^3^UniCamillus, International University of Health and Medical Science, Rome, Italy

## Abstract

Sjögren's syndrome (SS) is a chronic autoimmune condition characterized by autoantibody production, sicca syndrome, and periepithelial lymphocytic lesions in target tissues. A predisposing genetic background is likely, and, to date, several polymorphisms in non-HLA genes have been explored with interesting results. We investigated the association between the *STAT4*, *TRAF3IP2*, *HCP5*, and *IL10* polymorphisms and SS susceptibility and their possible role in the modulation of clinical and laboratory features. 195 consecutive patients with SS were enrolled and clinical and laboratory data were collected. 248 age- and sex-matched healthy subjects were used as controls. Genotyping was performed by allelic discrimination assays. A case-control association study and a phenotype-genotype correlation analysis were performed. A genetic risk profile was developed considering the risk alleles. Both the variant alleles of rs7574865 in the *STAT4* gene and rs3099844 in the *HCP5* gene were significantly more prevalent in patients than in controls (OR = 1.91 and OR = 2.44, respectively). The variant allele of rs3024505 of *IL10* resulted to be a susceptibility allele (OR = 1.52), while the variant allele of rs1800872 seemed to confer a protective effect for the development of the disease (OR = 0.65). A risk genetic profile showed a higher probability to develop the disease in subjects with at least three risk alleles; subjects with 4 risk alleles were not observed in the controls. *HCP5* rs3099844 was associated with anti-SSA (*P* = 0.006, OR = 3.07) and anti-SSB (*P* = 0.005, OR = 2.66) antibodies, severity of focus score (*P* = 0.03, OR = 12), and lymphoma development (*P* = 0.002, OR = 7.23). Patients carrying the *STAT4* rs7574965 variant allele had a higher risk of monoclonal component and leukopenia (*P* = 0.002, OR = 7.6; *P* = 0.048, OR = 2.01, respectively). We confirmed the association of SS with the *STAT4* and *IL10* genes and we describe a novel association with *HCP5*. In particular, we describe an association of this specific SNP of *HCP5* not only with disease development but also with autoantibody production and focus score suggesting a potential contribution of this variant to a more severe phenotype.

## 1. Introduction

Sjögren's syndrome (SS) is a systemic autoimmune condition characterized by a chronic inflammatory reaction in the exocrine glands [1]. Periepithelial lymphocytic lesions are characteristically present in the SS salivary glands, and the “activated” epithelium is known to contribute to the development, maintenance, and progression of the local autoimmune responses [1].

The presence of a predisposing genetic background has been suggested, and different environmental agents act as triggers of the disease [1]. Indeed, latent viral infections harbouring salivary glands are causally implicated in epithelium activation [1], and the persistence of viral genetic material seems to be able to alter epithelial cell biologic properties with consequent overexpression of type I IFN-inducible genes: the “*IFN signature*” [2]. The increased production of IFN provides protection from viral infections and drives the transcription of hundreds of genes implicated in antiviral response [3]. Moreover, IFN production in the SS salivary glands has been recently linked to endogenous retroviral element expression, normally silent [4]. Although in SS, a type I IFN signature has been proven both in the minor salivary glands (MSG) [5] and in peripheral blood cells [6]; a predominant type II IFN (IFN*γ*) production at tissue level compared to peripheral blood has been recently demonstrated [2, 7]. In the salivary glands, the main source of IFN*γ* is represented by CD4^+^-infiltrating cells (Th1 cells) which are also in control for the production of other cytokines including IL-2 and IL-10 [8].

Compared to healthy subjects, higher serum levels of IL-10, also correlated with autoantibody production, have been detected in SS [9]. In addition, elevated levels of this cytokine seem to be present in patients' saliva with evidence of a positive correlation with disease activity [10]. However, the role of this cytokine in SS pathogenesis is still not clear. Given the evidence of an altered production of IL-10 in SS, polymorphisms in the *IL10* (interleukin 10) promoter have been investigated with controversial results [11–13].

To date, a broad spectrum of polymorphisms not related to *MHC* genes has been investigated in SS. Recently, Nezos and Mavragani classified three classes of genes whose polymorphisms are possibly implicated in disease pathogenesis: genes involved in the interferon (IFN) pathway, genes involved in B cell function, and genes involved in the NF-*κ*B pathway [14].

Concerning the IFN pathway, a specific polymorphism (rs7574865) of *STAT4* (signal transducer and activator of transcription 4) seems to be associated with SS [15, 16] with evidence of a major risk in the homozygote variant [17]. Later on, other variants in the same gene appeared not only associated with SS but also with the increased expression of several IFN-inducible genes [17]. Genome-wide association studies (GWAS) confirmed the involvement of *STAT4* in SS predisposition [18–20].

The TRAF3-interacting protein 2 (*TRAF3IP2*) gene, also known as Act1, encodes for a protein known to be a negative regulator of B cell responses (by its interaction with CD40L and BAFF signalling) as well as a positive regulator of IL-17 signalling [21]. As these pathways are crucial in SS [22], polymorphisms of this gene might play a role in the disease susceptibility. Interestingly, mice deficient in Act1 develop systemic autoimmune disease with histological and serological features of human SS in association with systemic lupus erythematosus- (SLE-) like nephritis [23].

In our previous works, we described associations between SNPs in the *STAT4*, *IL10*, *TRAF3IP2*, and *HCP5* (HLA complex P5) genes and systemic lupus erythematosus (SLE) susceptibility [24–26]. Concerning *HCP5*, of note is the association that came out with the production of anti-Ro-SSA antibodies [25].

Taken all together, these considerations lead us to hypothesize a role of these polymorphisms in SS too.

For this purpose, we aimed to evaluate the association of polymorphisms in the *STAT4* (rs7574865), *TRAF3IP2* (rs33980500), *HCP5* (rs3099844), and *IL10* (rs1800872 and rs3024505) genes with SS susceptibility and to elucidate their role in the modulation of clinical and laboratory features in a cohort of Italian patients.

## 2. Materials and Methods

### 2.1. Sample Collection

One hundred ninety-five consecutive patients with SS (diagnosed according to the American-European Consensus Criteria) [27] were enrolled from our dedicated Sjögren's Clinic (Sapienza University of Rome). Study protocol included complete physical examination and blood drawing. The clinical and laboratory data were collected in a standardized, computerized, and electronically filled form including demographics, past medical history with date of diagnosis, comorbidities, and previous and concomitant treatments. The evaluation of clinical and laboratory parameters was assessed with a dichotomous score (present = 1; absent = 0). Written informed consent was obtained from each patient and the ethical committee of Sapienza University of Rome approved the study design. Two hundred forty-eight age- and ethnicity-matched healthy subjects, enrolled at the University of Rome Tor Vergata, served as controls. Demographic and clinical characteristics of the patients are reported in Table 1. Peripheral blood samples from all patients and controls have been collected and stored at -20°C until usage.

### 2.2. Clinical and Laboratory Data

Clinical and laboratory data were collected on a dedicated electronic support. Data related to a minor salivary gland biopsy (i.e., focus score (FS) or presence/absence of germinal centers (GCs)) were recorded. For each patient, the following information were recorded: sex, age at onset, age at diagnosis, presence of xerophthalmia, xerostomia, history of gland swelling, arthritis, and lymphoma. Patients' demographic and clinical findings are shown in Table 1.

Regarding the laboratory exams, the following parameters were evaluated: complete cell blood count, including the erythrocyte, leucocyte, and platelet counts; serum protein electrophoresis; serum levels of complements C3 and C4; antinuclear antibodies (ANA) and anti-SSA and anti-SSB antibodies; rheumatoid factor; and presence/absence of cryoglobulins. Each of these tests had to be positive in at least 2 occasions all along patients' clinical history to be considered positive. ANA were determined by means of indirect immunofluorescence (IIF) on Hep-2 and were considered present at a titer 1 : 160; anti-SSA and anti-SSB antibodies were determined by ELISA considering titers above the cut-off of the reference laboratory; the rheumatoid factor was determined by the Waaler-Rose test and/or Ra test; hypergammaglobulinemia and monoclonal component were determined by serum protein electrophoresis and, in case, serum and urine immunofixation; cryoglobulins were determined by cryoprecipitate detection (samples kept at 37°C, warm centrifugation, warm cell precipitation, serum conservation at 4°C, and cryoprecipitate detection after 7 days). Patient laboratory findings are shown in Table 1.

### 2.3. DNA Extraction and Genotyping

Genomic DNA was isolated from peripheral blood mononuclear cells using a Qiagen blood DNA mini kit. We chose to analyze polymorphisms in four genes involved in immune response and inflammation that were previously associated with a higher risk to develop SLE. The selected SNPs were the following: rs7574865 (*STAT4*, chromosome 2), rs3099844 (*HCP5*, chromosome 6), rs33980500 (*TRAF3IP2*, chromosome 6), and rs1800872 and rs3024505 (*IL10*, chromosome 1). Genotyping was performed by allelic discrimination assays by TaqMan technology (Applied Biosystems, Foster City, CA, USA) and real-time PCR. Each assay was run with positive (samples previously confirmed by direct sequencing as heterozygous and/or variant homozygous) and negative controls.

### 2.4. Statistical Analysis

The Hardy–Weinberg equilibrium was verified for all SNPs by Pearson's *χ* ^2^ test. Differences in alleles and genotype frequencies between cases and controls were evaluated by Pearson's *χ* ^2^ test. Odds ratios (ORs) with 95% CI were calculated. The cut-off for statistical significance was *P* < 0.05. A genotype-phenotype correlation analysis has been performed comparing cases with and without specific manifestations and considering the heterozygotes and variant homozygotes together (1 degree of freedom (df)). All statistical analyses were performed by the SPSS program ver. 19 (IBM Corp., Armonk, NY, USA).

### 2.5. Haplotype Analysis

Haplotypes were inferred using Arlequin, version 3.5 [28]. Differences in the haplotype distribution between cases and controls were evaluated by *χ* ^2^ test.

## 3. Results

Clinical and laboratory data are presented in Table 1 and are in line with previous cohorts [29]. As expected, the presence of lymphoproliferative complications was quite rare with evidence of non-Hodgkin lymphoma (NHL) only in nine cases (4.6%). Specifically, 7/9 cases presented with the typical MALT in the major salivary glands while the other two experienced a nodal and splenic NHL localization, respectively.

Polymorphisms in the *STAT4*, *HCP5*, *TRAF3IP2*, and *IL10* genes were analyzed in 195 patients with SS and 248 healthy controls. Deviations from the Hardy-Weinberg equilibrium were not observed for all studied polymorphisms. The distribution of genotype and allele frequencies and the comparisons between cases and controls are presented in Table 2. Polymorphisms in the *STAT4*, *HCP5*, and *IL10* genes are associated with SS susceptibility. Both the variant alleles of rs7574865 in the *STAT4* gene and rs3099844 in the *HCP5* gene were significantly more prevalent in patients than in controls, with an OR = 1.91 (95% CI 1.41-2.59) and *P* = 3 × 10^−5^ for *STAT4* and OR = 2.44 (95% CI 1.49-3.99) and *P* = 3 × 10^−4^ for *HCP5*, respectively.

We also observed an allele additive effect for *STAT4* SNP: indeed, patients carrying one variant allele had an OR = 1.35 (*P* = 0.15), while patients carrying two variant alleles had an OR = 6.79 (*P* < 0.0001). Regarding the *HCP5* SNP, it is interesting to note that the only two subjects with a homozygous variant genotype were present in the case group.

Concerning the *IL10* gene, the genotypic frequencies of both analyzed polymorphisms (rs3024505 and rs1800872) were significantly different between SS patients and controls: the variant allele of rs3024505 resulted to be a susceptibility allele (OR = 1.52), while the variant allele of rs1800872 seemed to confer a protective effect for the development of the disease (OR = 0.65).

We have inferred the haplotypes between the two *IL10* polymorphisms and between the rs3099844 and rs33980500 SNPs since the *HCP5* and *TRAF3IP2* genes are located on the same chromosome. However, the haplotype analysis did not improve the significance of the single locus association (see Supplementary Table S1).

**Figure 1:**
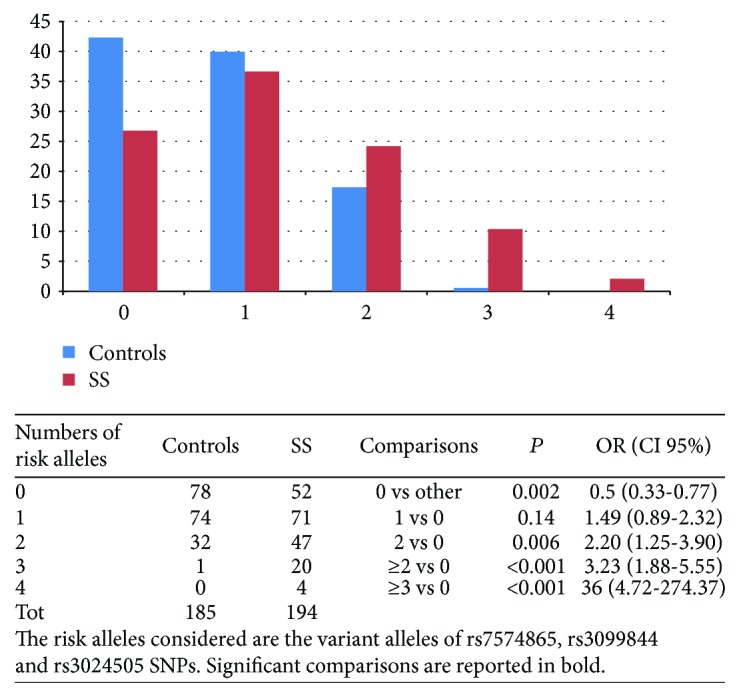
Counting of risk alleles in cases and controls.

Moreover, we counted the total number of risk alleles in each subject, considering as risk alleles the allelic variant of rs7574865 (*STAT4*), rs3024505 (*IL10*), and rs3099844 (*HCP5*) SNPs (we counted two risk alleles for homozygous variant genotypes). Then, we compared the risk allele number distribution between cases and controls (Figure 1). As expected, the class with no risk allele was significantly more prevalent in controls than in patients (*P* = 0.002, OR = 0.5). On the contrary, classes with 2 or more risk alleles are significantly more represented in cases than in controls (*P* < 0.001, OR = 3.23). In particular, subjects with at least three risk alleles have a higher probability to develop the disease (OR = 36, *P* < 0.001) and the class with 4 risk alleles is observed only in cases and never in the controls.

We further performed a genotype-phenotype correlation analysis to evaluate if these SNPs contribute to the modulation of clinical phenotypes (significant results are reported in Table 3; for a complete report of the evaluations, see Supplementary Table S2). Patients carrying the *STAT4* rs7574865 variant allele have a higher risk to develop the monoclonal component and leukopenia (*P* = 0.002, OR = 7.6; *P* = 0.048, OR = 2.01, respectively), while patients carrying the variant allele of *TRAF3IP2* rs33980500 were less predisposed to develop anti-SSB (OR = 0.4 and *P* = 0.043). Interestingly, the variant allele of *HCP5* SNP is associated with a higher risk to develop anti-SSA (OR = 3.07, *P* = 0.006), anti-SSB (OR = 2.66, *P* = 0.005), rheumatoid factor (OR = 2.17, *P* = 0.028), hypergammaglobulinemia (OR = 2.54, *P* = 0.007), leukopenia (OR = 2.1, *P* = 0.047), and lymphoma (OR = 7.23, *P* = 0.002). This result is particularly important considering the severity of this clinical manifestation: patients carrying the A allele have a significantly increased risk to develop lymphoma. Moreover, the variant of *HCP5* also is associated to the severity of the salivary gland FS (*P* = 0.03 and OR = 12) (Table 4).

## 4. Discussion

In this study, we confirm the association of SS with the studied polymorphism of *STAT4* and we show for the first time an association with a specific variant of the *HCP5* gene. Moreover, we describe that two variants in the *IL10* gene are associated with susceptibility to SS, with a risk (rs3024505) and a protective (rs1800872) effect, respectively. Both variants in the *HCP5* and *STAT4* genes were associated with a higher risk to develop a specific clinical phenotype. No association between *TRAF3IP2* SNP and SS came out; on the contrary, it seems to confer a protection towards the production of anti-SSB antibodies. An interesting result of our work is the possibility to define, with only three SNPs, a genetic profile model showing that the risk to develop the disease increases considerably with the number of risk alleles. In particular, subjects with at least three risk alleles have up to 36 times higher probability to develop the disease and subjects with 4 risk alleles are only present in cases and never observed in controls.

Considering the known involvement of STAT4 in the “*IFN signature*,” the observed association between rs7574865 polymorphism and SS is not surprising [30]. *STAT4* SNPs have been already described in other autoimmune conditions such as rheumatoid arthritis and SLE [31], and an association between the *STAT4* rs7574865 and the susceptibility to SS has been reported too [15, 18–20, 32]. Several studies observed a correlation between the minor allele of rs7574865 and higher levels of STAT4 mRNA [33, 34] and with increased sensitivity to IFN-alpha signalling [35]. These data could explain how the minor allele of rs7574865 confers a higher risk of developing SS. However, this SNP is located on the third intron of the *STAT4* gene and the precise mechanism that leads to the higher expression of STAT4 remains unclear. As in SS, the IL-17 axis gives a great contribution to the development and maintenance of the local inflammatory process [36]; it is also relevant to remind the known implication of STAT4 in Th17 response [37, 38]. According to this background, the association that we found between a *STAT4* SNP and the aberrant production of autoantibodies, cryoglobulins, and monoclonal component could be more easily explained.

Our study also describes for the first time an association between SS and a specific polymorphism of *HCP5*. In our previous work, this variant was found associated with the susceptibility to SLE and a very strong association was observed with the presence of anti-SSA antibodies [25]. This association between *HCP5* and anti-SSA antibodies also emerged in our SS cohort in which the rs3099844 variant allele appears to be associated with anti-SSB antibodies and RF as well. It is likely that *HCP5* confers a risk to a more aggressive inflammatory pattern characterized by a broader autoantibody production. Indeed, the salivary glands in patients with SS seem to represent the main inflammatory site in which the exposition of autoantigens takes place [39, 40]. Although the role of autoantibodies in the pathogenesis of SS is still controversial, their production mirrors the severity of the inflammatory process at the tissue level. Indeed, it is known that anti-SSA antibodies are linked with the presence of a more severe inflammatory process [40]. The presence of a FS > 1 is, in fact, strongly associated with anti-SSA and anti-SSB antibodies, and in general, the presence of these antibodies seems to provide a 9-fold higher risk to have a FS > 1 compared to seronegative patients [41]. Higher FS also correlates with the presence of GCs and systemic manifestations [40]; the association between GCs and lymphoma development is actually a matter of debate [42, 43]. Considering the severity of this complication, the identification of predictive genetic biomarkers is important for patient monitoring. Indeed, till now, very few studies have investigated the genetic involvement in lymphoma in SS, and only few associations have been identified, e.g., with the *MHTFR* [44], *BAFF* [45], and *TNFAIP3* [46] genes. In our study, the association, although very preliminary, of *HCP5* variant with lymphoma development is a promising result. Overall, the association between *HCP5* rs3099844 SNP and specific laboratory features (autoantibodies and leukopenia) as well as with the severity of the FS leads to hypothesize a role of this variant in a more aggressive pattern of disease. The SNP has been previously associated to other conditions such as Stevens-Johnson syndrome and toxic epidermal necrolysis [47] as well as sclerosing cholangitis [48] in which anti-SSA antibodies have been described in up to 11% of cases. Moreover, a genome-wide study identified an association between this variant and cardiac manifestation in neonatal lupus that is known to be associated with anti-SSA antibodies [49]. A different polymorphism (rs2395029) of *HCP5* was associated to psoriasis and psoriatic arthritis [50].

Considering the analyzed *IL10* SNPs, for one of them (rs3024505), we found an association with SS. On the contrary, a protective effect was observed for the other variant (rs1800872). IL-10 is a pleiotropic cytokine able to suppress Th1 cell response, to downregulate *MHC* class II antigens and costimulatory molecules on macrophages, and, given its ability to enhance B cell survival and proliferation, to induce the production of immunoglobulin and autoantibody [51]. The *IL10* gene is located on chromosome 1, and several polymorphisms have been identified [52] with possible consequences on its production [53]. The different outcomes that we observed between these two polymorphisms in SS might have possible implication in the regulation of IL-10 production. In particular, the rs1800872 is localized in the promoter region; the variant allele could have a protective role for the development of SS because it is associated with increased IL-10 production [54], which modulates the inflammatory response. However, the role of IL-10 in SS is still not clear.

Since in murine models a deficiency of the Act1 protein is able to determine a SS phenotype [23], we also investigated the *TRAF3IP2* gene. However, no contribution of *TRAF3IP2* gene polymorphism emerged in our study.

Although our cohort of patients is well characterized and accurately diagnosed, the limit of this study is represented by the small sample size. In particular, patients displaying a particularly severe pattern of disease were quite rare with only few cases experiencing lymphoproliferative complications. However, our results appear promising and possibly useful in identifying patients more prone to develop an aggressive disease. These data should be considered preliminary ones and a replication study is strongly recommended.

## 5. Conclusion

We confirm the associations between SNPs in the *STAT4* and *IL10* genes and SS susceptibility and we provide a novel association with a specific polymorphism in the *HCP5* gene. We also show how the genotyping of only three SNPs may allow to define a genetic risk profile for SS development.

Moreover, we also confirm the association between *HCP5* and the production of anti-SSA antibodies, and we further observe an association with other SS autoantibodies and with the FS. Taken all together, these associations might suggest a more aggressive pattern of disease in patients presenting the analyzed *HCP5* polymorphism. Our findings deserve further confirmation in larger cohorts and in populations of other ethnicities.

## Figures and Tables

**Table 1:** Clinical and laboratory data of the 195 SS patients.

Clinical features	
Sex (M/F)	9/186
Age (mean ± standard deviation, years)	58.1 ± 11.2
Age at diagnosis (mean ± standard deviation, years)	51.9 ± 11.1
Xerophthalmia	179/195 (91.7%)
Xerostomia	174/195 (89.2%)
Salivary gland swelling	44/195 (22.5%)
Arthritis	22/195 (11.2%)
Lymphoma	9/195 (4.6%)
Laboratory features	
ANA	168/195 (86.1%)
Anti-SSA	126/195 (64.6%)
Anti-SSB	86/195 (44.1%)
Hypergammaglobulinemia	67/195 (34.3%)
Rheumatoid factor	67/195 (34.3%)
Leukopenia	44/195 (22.5%)
Hypocomplementemia	21/195 (10.7%)
Monoclonal component	16/195 (8.2%)
Cryoglobulins	8/195 (4.1%)

ANA were considered positive if titer ≥ 1 : 160; anti-SSA, anti-SSB, and RF were considered positive according to the cut-off of the reference laboratory; hypergammaglobulinemia was diagnosed if total Ig ≥ 20% of total proteins; leukopenia was diagnosed if WBC < 4000/mm^3^; hypocomplementemia was diagnosed if C3 < 80 mg/dl and/or C4 < 15 mg/dl.

**Table 2:** Case-control association study.

*STAT4* rs7574865		SS (*N* = 195)	Controls (*N* = 243)	*P*	OR (95% CI)
Genotypes	GG	92	150	**0.002**	1.81 (1.23-2.65)
	GT^∗^	78	87		
	TT^∗∗^	25	6		
Alleles	G	262	387	**0.00003**	1.91 (1.41-2.59)
	T	128	99		

*HCP5* rs3099844		SS (*N* = 195)	Controls (*N* = 248)	*P*	OR (95% CI)
Genotypes	CC	149	221	**0.0003**	2.53 (1.5-4.24)
	CA	44	27		
	AA	2	0		
Alleles	C	342	469	**0.0003**	2.44 (1.49-3.99)
	A	48	27		

*TRAF3IP2* rs33980500		SS (*N* = 195)	Controls (*N* = 218)	*P*	OR (95% CI)
Genotypes	CC	167	183	0.63	0.88 (0.51-1.5)
	CT	28	34		
	TT	0	1		
Alleles	C	362	400	0.57	0.86 (0.51-1.44)
	T	28	36		

*IL10* rs1800872		SS (*N* = 194)	Controls (*N* = 278)	*P*	OR (95% CI)
Genotypes	CC	117	130	**0.004**	0.58 (0.40.0.84)
	CA	66	124		
	AA	11	24		
Alleles	C	300	384	**0.005**	0.65 (0.49-0.88)
	A	88	172		

*IL10* rs3024505		SS (*N* = 194)	Controls (*N* = 270)	*P*	OR (95% CI)
Genotypes	CC	137	209	0.1	1.43 (0.94-2.17)
	CT	48	58		
	TT	9	3		
Alleles	C	322	476	**0.025**	1.52 (1.05-2.21)
	T	66	64		

^∗^GT vs. GG: *P* = 0.15 and OR = 1.35; ^∗∗^TT vs. GG: *P* < 0.0001 and OR = 6.79; *P* = *p* value evaluated by Pearson's *χ* ^2^ test; OR (95% CI) = odd ratios with 95% confidence interval.

**Table 3:** Associations between disease phenotypes and studied polymorphisms.

Gene	Phenotype	*P*	OR (95% CI)
*HCP5 rs3099844*	Anti-SSA	**0.006**	3.07 (1.83-7.05)
	Anti-SSB	**0.005**	2.66 (1.32-5.36)
	Rheumatoid factor	**0.028**	2.17 (1.08-4.9)
	Hypergammaglobulinemia	**0.007**	2.54 (1.28-5.86)
	Leukopenia	**0.047**	2.1 (1-4.23)
	Lymphoma	**0.002**	7.23 (1.73-33.7)
*STAT4 rs7574865*	Monoclonal component	**0.002**	7.6 (1.68-34)
	Leukopenia	**0.048**	2.01 (1-4.07)
*TRAF2IP2 rs33980500*	Anti-SSB	**0.043**	0.4 (0.16-0.99)

**Table 4:** Association between *HCP5* SNP and severity of the focus score.

	Focus score	
HCP5 rs3099844	<3	≥3
AA	20	10
AC+CC	1	6

*P* = 0.03; OR = 12 (95% CI = 1.27-113.7).

## Data Availability

The data used to support the findings of this study are available from the corresponding author upon request.

## Supplementary Materials

Supplementary Materials Table S1: comparison of the haplotype distribution between SS patients and controls. Table S2: complete list of genotype/phenotype correlation analyses.
