# Supplementary material for: STAT4, TRAF3IP2, IL10, and HCP5 Polymorphisms in Sjögren's Syndrome: Association with Disease Susceptibility and Clinical Aspects
Source: J Immunol Res. 2019 Feb 10;2019:7682827. doi: 10.1155/2019/7682827 (PMC6387711; doi:10.1155/2019/7682827)
Supplement: Supplementary Materials — Table S1: comparison of the haplotype distribution between SS patients and controls. Table S2: complete list of genotype/phenotype correlation analyses. [file 7682827.f1.pdf]

Table S1. Comparison of the haplotypes distribution between SS patients and controls

TRAF3IP2/HCP5	**SS**	**Controls**	**Comparisons**	**P**	**OR (CI 95%)**
CC	319	329	CC vs others	**0.04**	0.67 (0.45-0.99)
TC	43	28	TC vs others	0.08	1.54 (0.94-2.55)
CA	23	18	CA vs others	0.48	1.25 (0.67-2.36)
TA	5	3	TA vs others	0.38	1.62 (0.39-6.85)
IL10*	**SS**	**Controls**	**Comparisons**	**P**	**OR (CI 95%)**
CC	232	301	CC vs others	0.35	1.14 (0.87-1.48)
CA	88	164	CA vs others	**0.006**	0.65 (0.49-0.89)
TC	66	63	TC vs others	**0.027**	1.52 (1.05-2.21)

*The haplotypes correspond to the following order of SNPs: rs1800872 (C>T) and rs3024505 (C>A)

Table S2. Complete list of genotype/phenotype correlation analyses

	STAT4 *RS7574865*		*HCP5* *RS3099844*		*TRAF3IP2* *rs33980500*		*IL10* *rs1800872*		*IL10* *rs3024505*	
**Phenotype**	**P**	**OR**	**P**	**OR**	**P**	**OR**	**P**	**OR**	**P**	**OR**
Xerostomia	0.86	0.92	0.46	0.68	0.90	0.92	0.44	1.60	0.58	0.76
Xerophthalmia	0.71	0.80	0.40	0.59	0.55	1.86	0.54	1.53	0.71	1.29
Anti-Ro/SSA	0.43	0.79	**0.006**	**3.07**	0.78	0.89	0.75	1.12	0.36	1.37
Anti-La/SSB	0.93	1.03	**0.005**	**2.66**	**0.043**	**0.40**	0.63	0.85	0.83	1.07
Anti-nuclear (ANA)	0.44	0.72	0.35	1.69	0.35	2.01	0.95	0.97	0.11	2.42
Rheumatoid factor	0.77	1.09	**0.028**	**2.17**	0.14	1.87	0.07	1.89	0.83	0.93
Hypergammaglobulinemia	0.74	1.11	**0.007**	**2.54**	0.82	0.90	0.67	1.16	0.49	0.79
Cryoglobulinemia	0.17	3.00	0.93	1.08	0.87	0.83	0.22	0.28	0.64	1.41
Monoclonal component	**0.002**	**7.61**	0.17	2.11	0.31	0.36	0.83	1.13	0.30	0.51
Hypocomplementemia	0.95	0.97	0.27	1.72	0.97	1.02	0.53	0.68	0.41	1.49
Leucopenia	**0.048**	**2.01**	**0.047**	**2.10**	0.91	0.95	0.95	0.97	0.96	0.98
Lymphopenia	0.23	1.78	0.32	1.70	0.30	0.45	0.55	1.33	0.97	1.02
Neutropenia	0.92	0.91	0.79	1.36	0.36	0.83	0.54	1.84	0.79	0.71
Lymphoma	0.53	0.78	**0.002**	**7.23**	0.78	0.74	0.85	0.85	0.30	2.02
Glandular swelling	0.80	0.91	0.42	1.15	0.34	1.55	0.65	0.82	0.95	1.02
Arhritis	0.21	1.77	0.36	1.56	0.62	1.34	0.97	0.98	0.93	1.04

Significant associations are reported in bold.
